# Fat-Associated Lymphoid Clusters in Inflammation and Immunity

**DOI:** 10.3389/fimmu.2016.00612

**Published:** 2016-12-21

**Authors:** Sara Cruz-Migoni, Jorge Caamaño

**Affiliations:** ^1^College of Medical and Dental Sciences, Institute of Immunology and Immunotherapy, University of Birmingham, Birmingham, UK

**Keywords:** lymphoid tissues, fat-associated lymphoid clusters, peritoneal inflammation, peritoneal immune responses, tertiary lymphoid structures

## Abstract

Fat-associated lymphoid clusters (FALCs) are atypical lymphoid tissues that were originally identified in mouse and human mesenteries due to that they contain a high number of type 2 innate lymphoid cells/nuocytes/natural helper cells. FALCs are located on adipose tissues in mucosal surfaces such as the mediastinum, pericardium, and gonadal fat. Importantly, these clusters contain B1, B2 and T lymphocytes as well as myeloid and other innate immune cell populations. The developmental cues of FALC formation have started to emerge, showing that these clusters depend on a different set of molecules and cells than secondary lymphoid tissues for their formation. Here, we review the current knowledge on FALC formation, and we compare FALCs and omental milky spots and their responses to inflammation.

## Introduction

The interactions between different types of immune cells are essential for both innate and adaptive immune responses to pathogens. Such interactions require strategically situated microenvironments to increase the chances that rare antigen-specific lymphocytes become activated. These specialized microenvironments are found in secondary lymphoid organs (SLOs) such as lymph nodes and Peyer’s patches. Specialized populations of CD45^−^ fibroblastic and endothelial cells express an arrangement of cell adhesion molecules, chemokines, and survival factors that guide the recruitment, co-localization and interactions of bone marrow-derived cells to their specific areas being T cells and dendritic cells (DCs) to the T cell area while B cells and follicular T helper cells to the B cell follicles. The development and organization of secondary lymphoid tissues is dependent on signals by the tumor necrosis factor (TNF) family of proteins such as lymphotoxin αβ (LTαβ), lymphotoxin β receptor (LTβR), and TNF-TNF receptor I and the downstream pathways such as activation of the nuclear factor kappa B family of transcription factors. The target genes of these pathways include the cell adhesion molecules VCAM-1, ICAM-1, MAdCAM-1, and the chemokines CXCL13, CCL19, and CCL21. Several detailed reviews of the processes that mediate lymph node development have been published (1–3).

Lymph nodes are characterized by well-defined B and T cell areas. B cell areas contain follicles that are organized by a specific population of reticular cell expressing the B cell-attracting chemokine CXCL13. Paracortical T cell areas are organized by a different type of stromal cells named T zone reticular cells that express CCL21 and CCL19, which attract T cells, and DCs to facilitate their interactions (4).

In addition to secondary lymphoid tissues such as lymph nodes, there are a series of inducible lymphoid tissues present in mucosal surfaces such as bronchial-associated lymphoid tissues (BALT) in the lung (5–9), gut-associated lymphoid tissues that comprise the isolated lymphoid follicles in the intestine (ILFs) (10–12), tear duct-associated lymphoid tissues (13, 14), nasopharyngeal-associated lymphoid tissues (NALT) (15, 16) and portal tract-associated lymphoid tissues (17, 18) to cite a few examples (see Table 1). Recently a novel type of lymphoid tissue called fat-associated lymphoid clusters (FALCs) has been identified in the mesenteries of humans and mice (19).

**Table 1:** **Main characteristics of mucosal lymphoid tissues**.

Lymphoid structure	Location	Structural organization	Ontogeny	Developmental requirements	Reference
Bronchial-associated lymphoid tissue (BALT)	Near the basal side of the bronchial epithelium of the lungs	Arranged in a B cell follicle with clusters of IgD^+^ cells, grouped around follicular dendritic cells (FDCs). They also contain a discrete T cell zone	Formation after birth, following antigen exposure or inflammatory challenge. High frequency in the neonatal stage	Defective architecture in *Lta*^−^*^/^*^−^ mice. Defective number in *Il7ra*^−^*^/^*^−^ mice. Absent in mice lacking *Cxcl13, Ccl19*, and *Ccl21a*	Fleige et al. (5), Kocks et al. (8), Moyron-Quiroz et al. (20), Rangel-Moreno et al. (9)

Isolated lymphoid follicles	Along the anti-mesenteric wall of the small intestine	Composed of a B cell area (germinal center) and few T cells	Formation after birth in response to inflammation or infection	Absent in *Lta*^−^*^/^*^−^, *Ltbr*^−^*^/^*^−^ *Aly/aly, Cxcr5*^−^*^/^*^−^, and *Rorc*^−/−^ mice. Requirement of commensal flora for maturation	Baptista et al. (10), Hamada et al. (11), Lorenz et al. (12), Velaga et al. (21)

Nasopharyngeal-associated lymphoid tissue (NALT)	Nasal passages of the nasal cavity	Composed of B and T cell areas	Formation after birth, first detected at postnatal day 7 in mouse	Defective formation in *Lta*^−^*^/^*^−^, *Ltbr*^−^*^/^*^−^, *Il7ra*^−^*^/^*^−^, *Aly/aly, Cxcr5*^−^*^/^*^−^, and *Cxcl13*^−^*^/^*^−^ mice. Absent in *Id2*^−^*^/^*^−^ mice. Requirement of microbiota for maturation	Asanuma et al. (15), Fukuyama et al. (16), Harmsen et al. (22), Rangel-Moreno et al. (23)

Tear duct-associated lymphoid tissue (TALT)	Lacrimal sac and tear duct of the eye	Composed of B cell aggregates, DCs and T cells	Formation after birth, between postnatal days 5 and 10 in mouse	Defective size and number in *Lta*^−^*^/^*^−^, *Il7ra*^−^*^/^*^−^, and *Cxcl13*^−^*^/^*^−^ mice. TALT formation is preserved in *Id2*^−^*^/^*^−^ and *Rorc*^−/−^ mice	Nagatake et al. (13), Paulsen et al. (14)

Portal tract-associated lymphoid tissue	Portal triad of the liver	Composed of B cell aggregates, FDCs and T cells	Formation after birth, triggered in response to bacterial and viral infections	Formation may be dependent on CCL21 expression	Grant et al. (17), Yoneyama et al. (18)

## The Structure of FALCs

Fat-associated lymphoid clusters are non-classical lymphoid tissues associated to adipocytes in mucosal surfaces, including omental, mesenteric, mediastinal, gonadal, and pericardial fat (19, 24, 25).

The frequency of FALCs varies among different adipose tissues (AT) in mice. Whereas gonadal AT has as little as 1–2 clusters, the omentum can harbor up to 80 clusters per depot in homeostatic conditions (24). This heterogeneity is also reflected in FALC size, which ranges from 100 to 500 µm in diameter (19).

Unlike lymph nodes, FALCs are not encapsulated and are in direct contact with surrounding adipocytes (19). The arrangement of leukocytes found in FALCs also differs from the organization of conventional SLOs. For instance, no discernible B and T cell compartmentalization areas are evident. Instead, FALCs from mesenteric AT are composed of a tight cluster of B220^+^ or IgM^+^ B cells, with variable numbers of CD4^+^ T cells and CD11b^+^ myeloid cells (19, 24). Both myeloid cell precursors (CD31/ER-MP58^+^) and mature macrophages (F4/80^+^) have been detected in omental FALCs, suggesting that these lymphoid clusters form permissive microenvironments where the former cells can proliferate locally to be a source of free macrophages within the peritoneal cavity (32). A similar process is likely to take place in FALCs in the mediastinum and pericardium (33, 34). Importantly, FALCs contain type 2 innate lymphoid cells (ILC2) that can support the proliferation of B1 cells through the expression of IL-5 (19).

B cell recruitment to mesenteric FALCs requires the presence of a network of stromal cells expressing the chemokine CXCL13 (24). These cells are found scattered along the lymphoid clusters and are thought to be different from follicular dendritic cells (FDCs), which require signaling through LTβR to induce CXCL13 expression (35). Importantly, FALCs are present in *Cxcl13*^−/−^ mice although they are devoid of B cells and their size is smaller than in their wild type littermates.

Fat-associated lymphoid clusters are highly vascularized as shown by their close association to blood vessels (19, 24). Moreover, lymphoid clusters in the omentum have also been found to contain high endothelial venules (HEVs), a specialized type of post-capillary venules essential for lymphocyte trafficking (30, 36). In contrast, FALCs connection with lymphatic vessels remains to be further investigated (24, 25, 27, 37). Earlier evidence has shown that FALCs in the omentum can collect antigens and particles directly from fluids within the peritoneal cavity (27).

## Developmental Requirements for FALC Formation

Conventional SLOs such as lymph nodes and Peyer’s patches develop in a timely manner during embryogenesis, independently of pathogen-induced inflammation (38). In contrast, FALCs develop postnatally and could be identified at around 2–3 weeks after birth, reaching a plateau at around 18 weeks of age in mice (24). Unlike classical SLOs, FALC formation is not dependent on LTβR signaling as shown by the occurrence of these clusters in *Ltβr*^−^*^/^*^−^ and *Ltα*^−^*^/^*^−^ mice (24). Furthermore, FALCs also form in *Rag2*^−^*^/^*^−^ and *Rorc*^−^*^/^*^−^ mice, which lack B and T cells and LTi/ILC3 cells, respectively. FALCs are not the only lymphoid structures that develop independently of LTi cells and LTβR signaling, as other mucosal-associated lymphoid tissues, but also follow these developmental requirements (see Table 1) (9, 13, 16, 39).

**Figure 1:**
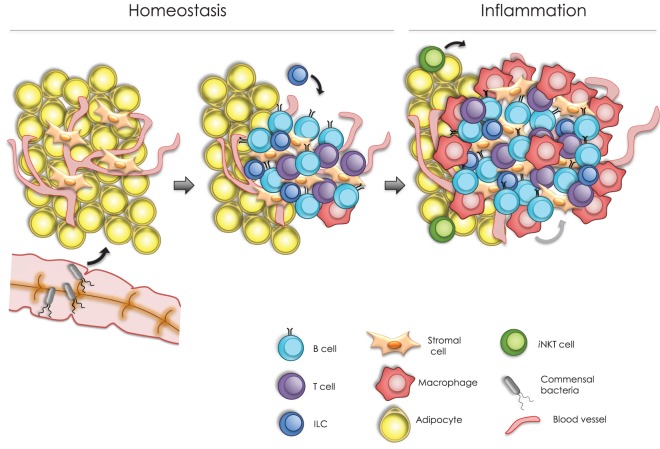
**Model of fat associated lymphoid cluster (FALC) formation**. In homeostasis, FALC formation can be modulated by different signals, including colonization by commensal microbiota, tumor necrosis factor (TNF) signaling, and type 2 innate lymphoid cells function. These signals may trigger cytokine production from local cells within the adipose tissue. For instance, CXCL13-expressing stromal cells can promote the recruitment of B cells into arranged lymphoid clusters. Following an inflammatory challenge, FALCs increase in number and size. Under these conditions, infiltrating macrophages are able to secrete TNF, which upon signaling through TNF receptors in stromal cells, promoting FALC growth. Moreover, invariant natural killer T cells stimulate FALC formation through the production of T helper type 2 cytokines, including IL-4 and IL-13, which may also promote the resolution of inflammation.

Fat-associated lymphoid clusters are absent in *Rag2*^−^*^/^*^−^*Il2rg*^−^*^/^*^−^ mice, which lack lymphocytes and ILCs, indicating a requirement of the latter for their development (24). Moreover, FALC formation is defective in *Tnfrsf1a*^−^*^/^*^−^*Tnfrsf1b*^−^*^/^*^−^ mice, which lack TNF receptors (TNFR1 and TNFR2) (24). Further analysis has shown that AT macrophages are the main source of TNF and that stromal cells respond to this cytokine *via* TNFR, leading to FALC formation (Figure 1) (24).

Fat-associated lymphoid cluster numbers are also greatly reduced in germ-free (GF) mice, suggesting a potential role of commensal flora in the formation of these structures (24). Interestingly, other lymphoid structures such as ILFs, which are found in the wall of the small intestine, are reduced or immature in GF mice (10). This suggests that similar to ILFs, the presence of commensal microbes or their by-products results in an inflammatory stimulus that induces the formation of FALCs. It will be interesting to test whether disruption of the intestinal epithelial barrier has an effect in the number and/or cellular composition of mesenteric FALCs (29).

## Are FALCs Similar to Milky Spots (MS) of the Omentum?

Milky spots were first described by von Recklinghausen in 1863 as white spots in the omentum of rabbits (40). These structures were later characterized as highly vascularized accumulations of macrophages, B and T lymphocytes, and mast cells within the stroma of the greater omentum (41, 42). The omentum is generally divided in two parts, the greater and lesser omentum, depending on its position within the peritoneal cavity. Moreover, the omentum contains two distinguishable regions: a translucent collagenous membrane-like region and an adipose-rich region containing blood and lymphatic vessels, stromal cells, and clusters of immune cells (43). In mice and humans, MS have been found in the adipose region of the omentum, whereas in dogs, the aggregates of lymphoid cells are only found scattered in the translucent area (43, 44). However, no clear distinction in the exact location of MS within the omentum has been established. Interestingly, some authors have divided MS in two categories: vascularized and non-vascularized MS. Vascularized MS are supplied with blood vessels and are found in the omental fat region. On the other hand, non-vascularized MS do not have blood supply and are located in the omental membrane (45). Whether these features that translate into functional differences remain to be investigated.

A close examination showed no discernible differences between MS and the FALCs in mesenteries, mediastinum, and gonadal fat (see Table 2). In light of their similar characteristics but distinct locations and for clarity, we will be calling the omental MS as omFALCs while mesFALCs correspond to the clusters in the mesenteries, medFALCs the structures in the mediastinum, perFALC for the structures in the pericardium, and gonFALC the clusters present in gonadal fat.

**Table 2:** **Comparison of milky spots (MS) and fat-associated lymphoid clusters (FALCs)**.

Feature	MS	FALCs
Location	Greater omentum	Mesenteric, mediastinal gonadal, and pericardial fat (19, 24, 25)

Size	349–756 µm in diameter in humans (26). 850 µm in diameter in healthy rabbits (27)	100–500 µm in diameter in mice (19). Size tends to increase with age (28)

Cell composition	Macrophages (47.5%)	B lymphocytes
	B lymphocytes (29.1%)	T lymphocytes
	T lymphocytes (11.7%)	Macrophages
	Mast cells (6.1%) (26)	
	Type 2 innate lymphoid cells (ILC2) (19)	ILC2 (20–40%) (19)
	CXCL13^+^ and FDCM1^+^ stromal cells (29, 30)	CXCL13^+^ stromal cells (24)

Developmental requirements	MS develop independently of ILC3/LTi cells and the chemokines CCL19 and CCL21. On the other hand, MS are defective or absent in *Cxcl13*^−^*^/^*^−^ and *Ltα*^−^*^/^*^−^ mice (30)	FALCs develop independently of ILC3/LTi cells and LTβR signaling. In contrast, their development is dependent on TNF signaling on stromal cells. IL-4R signaling and the presence of invariant natural killer T cells are also required. The requirement for type 2 ILCs in FALC development remains to be investigated (24)

Ontogeny	Accumulation of myeloid cells in the greater omentum has been observed at 20 weeks of gestation. True MS are observed at 35 weeks of gestation in humans (31)	Mesenteric FALCs are formed after birth, with visible clusters at 2–3 weeks of age in mice (24)

## FALC Respond to Inflammation and Immunization

MesFALCs and omFALCs increase in number and size in response to acute or chronic peritoneal inflammation (24, 27, 37). For instance, upon intraperitoneal (IP) injection of Zymosan, a yeast-derived ligand of Toll-like receptor 2, the abundance and size of mesFALCs increased significantly (24). *Rag2*^−^*^/^*^−^ and *Cd1d*^−^*^/^*^−^ mice failed to induce FALC formation in response to Zymosan-induced inflammation. Interestingly, IP injection of invariant natural killer T (iNKT) cells into *Rag2*^−^*^/^*^−^ mice was able to restore mesFALC formation following Zymosan challenge, indicating a role of iNKT cells in this process (24). More specifically, activated iNKT cells can produce T helper type 2 (T_H_2) cytokines, including IL-4 and IL-13, which may have redundant roles in the formation of FALCs following peritoneal inflammation in the BALB/c strain background (Figure 1) (24).

Likewise, IP injection of lipid A, a component of bacterial lipopolysaccharide, leads to an increase in the number of B1 cells and macrophages in the omentum (29). Moreover, IP injection of polydextran particles or polyacrylamide beads increases the number and size of omFALC (37, 46). Different models of inflammation including TNF injection, *Escherichia coli* infection, and cecal ligation lead to the influx of neutrophils to the omFALC *via* HEVs (36).

Earlier evidence of the mechanisms by which FALCs respond to immunization comes from studies on immune responses in the omentum. Using an elegant mouse model that lacks spleen, lymph nodes, and Peyer’s patches (SLP mice), Rangel-Moreno, Randall, and colleagues have shown that omFALC are sufficient to respond to an immunological challenge and support T cell responses, immunoglobulin switching, and moderate affinity maturation (30). It is possible that the antigen-specific immune response shown in serum in SLP mice upon IP immunization that had been attributed to the omFALC may have a relative contribution from mesFALCs. Peritoneal immunization resulted in a marked recruitment of macrophages to the omFALC similar to what has been shown in mesFALCs (24). OmFALCs were shown to collect IP injected fluorophore-labeled antigens or GFP-labeled tumor cells. Peritoneal cell migration to the omentum is mediated by mechanisms that are both dependent and independent of chemokines, but clustering of B and T lymphocytes is dependent on the latter.

Macrophages are the most important cell type to engulf and eliminate bacteria in omFALCs (30). Indeed, it has been shown in a diet-induced obesity model that macrophages in gonFALC are able to proteolytically process antigen and present it in the context of MHC II complexes, indicating that they can function as antigen-presenting cells to induce T cell proliferation (47). Furthermore, IP immunizations with T-cell-dependent antigens showed that antigen-specific B cells undergo expansion, Ig switching, and acquired markers of GC reaction further indicating that adaptive immune responses take place in mesFALCs (24).

Mesenteric FALCs are also able to respond to intestinal helminth infection (*Nippostrongylus brasiliensis*) *via* ILC2 activation and expression of the T_H_2 cytokines IL-5 and IL-13. These cytokines are produced in response to IL-33 and IL-25 and promote goblet cell hyperplasia and helminth expulsion (19, 48). Likewise, a recent report has shown that medFALCs and perFALCs respond to both lung infection with the nematode *Litomosoides sigmodontis* and lung inflammation *via* fungal allergen inhalation (33). Under these conditions, medFALC stromal cells produce IL-33, which leads to ILC2 activation and IL-5 secretion. This in turn promotes the recruitment and activation of B cells and culminates in the production of natural IgM antibodies (Ab) for local protection (33).

A recent report has shown that medFALC are present in large numbers in two animal models of autoimmunity that develop a phenotype similar to human systemic lupus erythematosus (SLE) (49). The MRL/MpJ-lpr mouse model carries a mutation in the gene encoding Fas, a cell membrane receptor that induces caspase activation and apoptosis. This mutant strain develops a severe autoimmune syndrome with lymphadenopathy, splenomegaly, and the production of anti-double strand DNA (dsDNA) Ab as well as immune complex deposition in several organs including kidney, resembling human SLE. The BXSB/MpJ-Yaa mice developed systemic autoimmunity, with males being more affected due to a locus called autoimmune accelerator located in the Y chromosome. Both strains showed large medFALCs size that correlated with a significant increased level of immune cell infiltration in the lungs with respect to their control strains (49). It will be interesting to assess whether medFALCs in the MRL/MpJ-lpr model contained anti dsDNA Ab-forming B cells.

Several reports indicate a link between FALCs and cancer metastasis in the peritoneum. A study has shown a direct correlation between the presence and number of FALCs and the colonization of the clusters by ovarian cancer cells, with the omentum containing the largest number of FALCs and metastasis/foci of tumor cells (50). The foci formation by tumor cells in FALCs is not affected by the absence of lymphocytes, indicating a role for specialized stromal cells, endothelium, and adipocytes in the clusters that facilitate malignant cell recruitment. Similarly, IP injection of gastric and colon cancer cells resulted in their rapid migration and survival in omental and mesFALCs (51–53). These reports together with the evidence of metastasis formation in the omentum and peritoneal cavity for some human cancers (54) indicate the presence of a favorable microenvironment in FALCs that allows the tumor cells to thrive and form micrometastasis in these tissues. Along this line, some reports have shown that gastric, colon, and some liver cancer cells express the chemokine receptors CCR4 and CXCR4, whereas cell populations from omFALCs and mesenteries can express their ligands (CCL22 and CXCL12), which may favor their migration to these structures (51, 52, 55).

## Conclusion

The rapid formation of FALCs following inflammation or infection, the changes in cellular composition and the presence of innate immune cells and B cells undergoing a germinal center reaction indicate a central role for these clusters in the formation of local immune responses.

Understanding what signals and cells are essential to FALC formation in homeostasis and following infection will allow inducing their formation to support antitumor responses. Conversely, it may be possible to reduce or preclude FALC formation during aberrant immune responses or peritoneal inflammation.

The physiological significance of the close association of FALCs with visceral AT remains to be further investigated. It is tempting to hypothesize that FALCs have an additional function in supporting type 2 ILCs and other immune cells that ultimately maintain the homeostasis of AT and whole body metabolism. Ultimately, understanding FALC formation in the mesenteries, omentum, and gonadal fat may facilitate the design of therapies to target low-grade chronic inflammation and other symptoms associated with obesity and metabolic syndrome.

## Author Contributions

SC-M prepared the tables and figure. SC-M and JC wrote the manuscript.

## Conflict of Interest Statement

The authors declare that the research was conducted in the absence of any commercial or financial relationships that could be construed as a potential conflict of interest.
